# Soft-Tissue Augmentation around Dental Implants with a Connective Tissue Graft (CTG) and Xenogeneic Collagen Matrix (CMX)—5-Year Follow-Up

**DOI:** 10.3390/jcm12030924

**Published:** 2023-01-24

**Authors:** Jakub Hadzik, Artur Błaszczyszyn, Tomasz Gedrange, Marzena Dominiak

**Affiliations:** 1 Department of Dental Surgery, Faculty of Medicine and Dentistry, Medical University of Wrocław, 50-367 Wrocław, Poland; 2 Department of Orthodontics, TU Dresden, 01069 Dresden, Germany

**Keywords:** dental implant, TKT, soft tissues, CTG, connective tissue, CMX, tissue graft

## Abstract

Proper horizontal and vertical thickness of the gingival connective tissue has been proven to be one of the success criteria in dental implant and reconstructive surgery. When thin tissue is found, gingiva augmentation methods can be used to increase the quality and volume of the tissue. Many methods have been described, among them pedicle soft-tissue flaps or autogenic tissue grafts. As an alternative to patients’ own tissue, xenogenic materials can be used for grafting. The fundamental issue is to choose a material that will ensure the maximum therapeutic effect, while also minimizing the negative influence on the patient’s health. The aim of this study was to compare gingival augmentation procedures using a palatal connective tissue graft (CTG) and an xenogenic soft-tissue substitute, Geistlich Mucograft (xenogeneic collagen matrix; CMX), and assess whether the timing of the graft surgery influences the clinical outcomes. The original study was a randomized control trial with a total of 75 implants placed. The patients received the soft-tissue thickening 3 months before the implant placement or 3 months after the implant placement (depending on the group). A connective tissue graft (CTG) or Geistlich Mucograft were used (depending on the group). For both the CTG and Geistlich Mucograft, better clinical outcomes were observed for maintaining the alveolar bone level and the thickness of the attached gingiva compared to the control group with no gingival augmentation. The Geistlich Mucograft showed good clinical performance in comparison to the control. Soft-tissue augmentation with the CTG before the implant placement was found to be most efficient method in terms of a stable increase of the tissue thickness since, throughout the entire observation period, the greatest increase of 1.035 mm (SD = 0.73 mm) in thickness was observed. Statistically important differences in the tissue thickness baseline compared after 5 years were observed for groups G1 vs. G2b (no augmentation vs. CTG before), G1 vs. G3b (no augmentation vs. CTG after) and for groups G2b vs. G3a (CTG before vs. CMX after).

## 1. Introduction

The proper width and thickness of the gingival connective tissue has been proven to be one of the success criteria in dental reconstructive surgery. Good quality soft tissue determines the possibility not only to obtain full primary wound closure during the bone defect reconstruction, but also assures effective implant treatment. Moreover, it is very important to assure the proper emergence profile of the implant supported restoration, which gives the opportunity to achieve the highest aesthetic appearance. In the long-term, the correct width and thickness of the attached keratinized tissue is the key point to ensure the stable position of the gumline around the implant’s neck. Linkevicius [1] found that the initial gingival tissue thickness at the crest may significantly affect the marginal bone loss (MBL) around implants. They also found that, when the soft tissue was less than 2.0 mm thick, more crestal bone loss appears, which is in agreement with other studies [2,3]. Thick soft tissue helps to prevent the development of an inflammatory reaction and clinical attachment-level loss, as well as alveolar bone loss. Besides, it prevents gingival recessions arising, which have an influence on the final aesthetics and on the likelihood of developing inflammatory complications. There are many keratinized gingiva augmentation methods using pedicle soft-tissue flaps [4] or autogenic tissue grafts [5]. Both have many modifications and can be applied to different treatment techniques, including on-lay or in-lay techniques. However, connective tissue graft-harvesting carries an additional burden for the patient, mainly in the form of a second operating site and the pain this entails. Alternative xenogeneic materials also exist. The fundamental issue is to choose the material which will assure the maximum therapeutic effect while minimizing the negative influence on the patient’s health. In a previous study by Puzio et al. [6,7] comparing palatal CTG (connective tissue grafts) with the porcine collagen matrix, Geistlich Mucograft (CMX; xenogeneic collagen matrix), it was shown that the thicker the soft tissue, the less MBL is observed, determining 2.88 mm as the critical value for the keratinized soft-tissue thickness TKT (TKT = thickness keratinized tissue). Furthermore, the authors concluded that, in the case of a thin soft-tissue biotype, gingival augmentation should be done 3 months before the implant placement [6,7]. Puzio et al. [6,7] found a CTG superior to an xenogeneic matrix. However, the Geistlich Mucograft was found to perform well and offers an alternative method to connective tissue grafts in gingival augmentation procedures.

The aim of this study was to perform a long-term, 5-year observation follow-up study.

The hypotheses of this study were:

1. The augmentation procedure of gingiva does not improve the thickness of the soft tissues in the aesthetic area in a 5-year observation.
2. The non-inferiority of the Geistlich Mucograft (Geistlich Pharma AG, Wolhusen, Switzerland) to the connective tissue graft in a gingiva augmentation procedure in a 5-year observation.

## 2. Materials and Methods

### 2.1. Study Design

The present study is a long-term observational study. The study was performed in the Wroclaw Medical University Dental Clinical and Teaching facility. The study protocol of the original randomized control trial (RCT) was approved by a local ethical committee (registration number No. KB-217.2012); the RCT was registered under the clinical trial registration number NCT04243460 (ClinicalTrials.gov). A total of 67 patients (42 women and 25 men) aged between 18 and 60 years participated in the original study; all the patients gave two written consents: the first was a general consent to have dental implants placed, and the other consent involved participation in the study. The study was conducted in full compliance with the Declaration of Helsinki. A protocol for this follow-up required a new bioethics committee approval that was granted (registration number 861/2021). The details on the specific inclusion and exclusion criteria, the exact clinical procedures and the 12-month data were reported in our previous publications [6,7].

#### Patients Randomization

**Figure 1:**
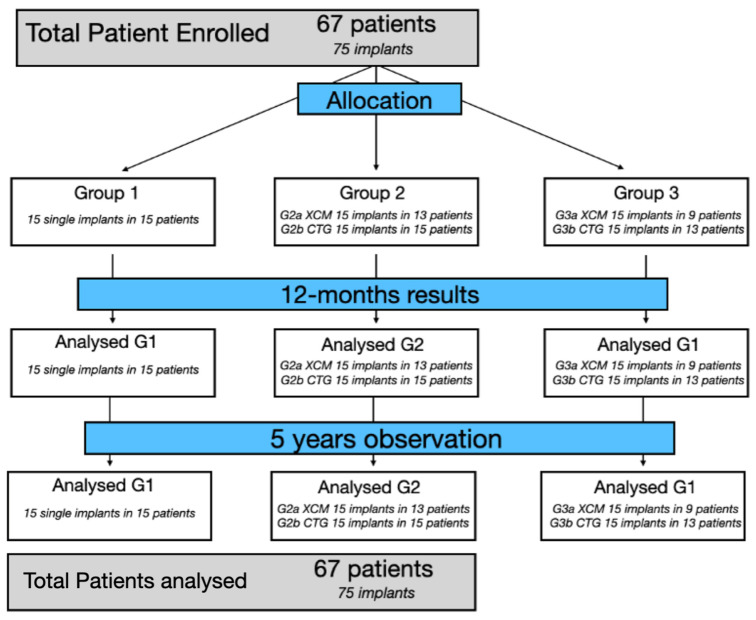
Flow chart, study schedule and timeline.

The details regarding the randomization and group allocation are outlined in the original study [7]. Briefly, 67 patients (42 women and 25 men), aged between 18 and 60 years (at the time of the surgery), were recruited according to a randomization protocol (double-blinded study protocol) and treated with a subepithelial connective tissue graft harvested from the palate or an xenogeneic collagen matrix, Geistlich Mucograft (Geistlich Pharma AG, Wolhusen, Switzerland). All the patients were divided into three groups, according to the gingival augmentation method, and are presented below and in Figure 1:

G1—No gingival augmentation. Fifteen single implants in fifteen patients.

Groups 2 and 3 were divided into two subgroups, according to the material used:

Group 2—Thickening of the soft tissue 3 months before the implantation as a preliminary pre-implantation intervention before the implant placement. Total 30 implants in 27 patients.

Group 2 consisted of two subgroups:

(a) G2a: CMX before Geistlich Mucograft, 15 implants in 13 patients (11 patients with single, 2 patients with double implants);
(b) G2b: CTG before a connective tissue graft from the palate (CTG); 15 implants in 14 patients (13 patients with single and 1 patient with double implants).

Group 3 involved thickening the soft tissue 3 months after the implantation; a total of 30 implants in 25 patients. 

Group 3 consisted of two subgroups:

(a) G3a: CMX after Geistlich Mucograft; 15 implants in 12 patients (9 patients with single, 3 patients with double implants)
(b) G3b: CTG with a connective tissue graft from the palate (CTG); 15 implants in 13 patients (11 patients with single, 2 patients with double implants).

A total of 75 tapered implants (Conelog, Camlog; CAMLOG Biotechnologies GmbH, Basel, Switzerland) were used in the study. The implant-loading took place after 6 months. All the implants were restored with metal-ceramic cemented crowns with semi-permanent cement (Implantlink, Detax, Ettlingen, Germany).

### 2.2. Inclusion and Exclusion Criteria

To qualify for the study, the patients had to be >18 years and have missing teeth in the aesthetic zone. The additional inclusion criteria were as follows:

- missing single or double teeth in the anterior area of their upper or lower jaw, with a proper interarch relationship (incisors, canines and first premolar), ridge width (bucco–lingual) greater than 5 mm at its narrowest point and minimum height of keratinized gingiva of 2 mm buccally;
- no previous soft-tissue augmentation procedure at the experimental site.

The exclusion criteria included previous grafting procedures performed in the area of interest and systemic or local diseases that could compromise healing or osteointegration. Smokers and patients with bruxism were also excluded from the study. The additional exclusion criteria were as follows:

- implants placed with an insertion torque of 35 Ncm or less;
- irradiation in the head and neck area; untreated periodontitis;
- poor oral hygiene (plaque score API 20%, bleeding score 10%);
- poor motivation;
- fresh post-extraction sockets.

### 2.3. Surgical Procedures

The surgical procedures were conducted under local anesthesia and are described in detail in the previous study [7]. In brief, 75 implants were successfully placed in 67 patients. The patients received soft-tissue thickening 3 months before the implant placement or 3 months after the implant placement (depending on the group); connective tissue or a Geistlich Mucograft were used (depending on the group).

### 2.4. Measurement of Marginal Bone Loss (MBL)

Intraoral radiographs were taken using the straight-angle technique with a holder (Visualixe HD, Gendex, Hatfield, PA, USA). The radiologic evaluation and measurements were performed using the RVG (Gendex, Hatfield, PA, USA). Before the calculation of the crestal bone changes, an RVG image was calibrated using the calibration in the Gendex software. The diameter of the implants was used as a reference point for the calibration. The same device and software were used for the 12-month and 5-year follow-ups.

### 2.5. Soft-Tissue Measurement

The TKT was measured using ultrasonography with a Pirop dental ultrasound device (Echoson Company, Puławy, Poland). For the purpose of this study, the results of the ultrasound examination conducted 12 months after the procedure were compared to the 5-year results. The measurements were made at two points (10 measurements at each point for each patient) with the use of ultrasound equipment (Pirop, Echoson), and the mean value for Points 1 and 2 was used for further calculations. Point 1 was considered to be in the middle of the line connecting the cemento-enamel junction (CEJ) to the adjacent teeth, and Point 2 was on the muco-gingival junction (MGJ).

### 2.6. Clinical Outcome

The clinical evaluation included bleeding on probing (BOP). The probing depths (PD) at four sites (mesial, distal, buccal, lingual) were used to evaluate the presence of peri-implantitis, according to Derks et al. [8], who define peri-implantitis as implants demonstrating bleeding on probing/suppuration and bone loss >2 mm. The implant survival rate was calculated as the number of implants that remained integrated throughout the 5-year follow-up period.

### 2.7. Follow-Up

**Figure 2:**
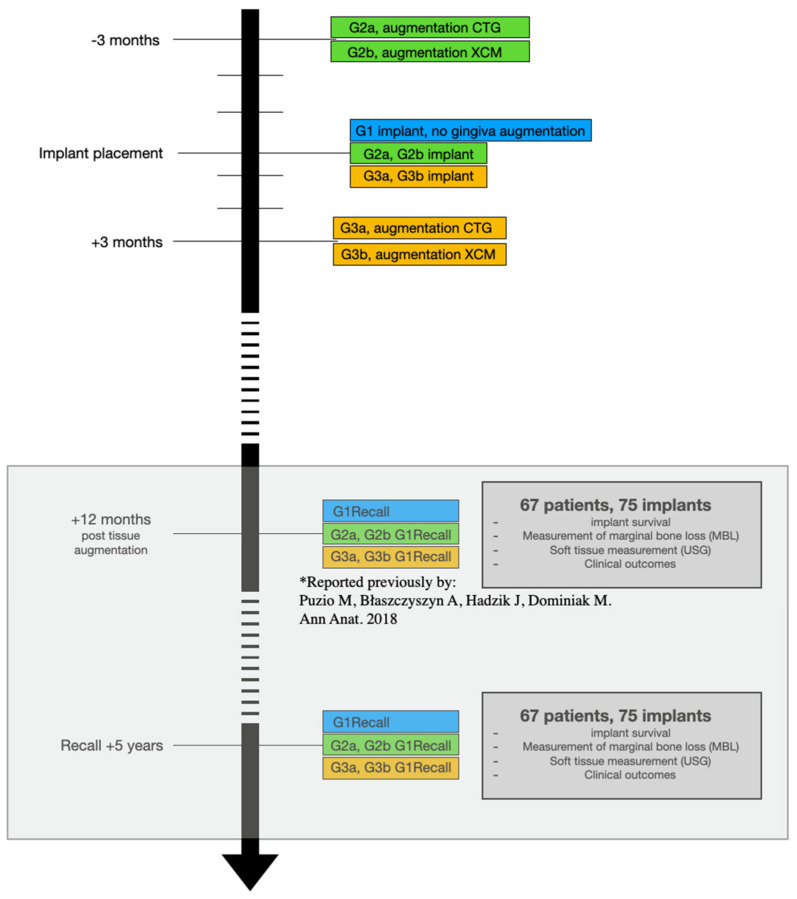
Study timeline, primary and secondary study endpoints [7].

After the original study had finished, most of the patients remained under regular maintenance in our clinic (Figure 2). Some of the patients had maintenance done in their place of residence for their convenience. No severe problems were reported by the patients during the 5-year period. All the patients were called for a free follow-up visit 5 years after the dental implant placement. A follow-up clinical appointment including clinical, radiological and USG evaluations was conducted by the same investigator (AB) who performed the postoperative measurements in the original study; this investigator was independent from the surgery team and did not know what material was used in each patient.

### 2.8. Primary and Secondary Endpoints

An assessment of the soft-tissue thickness was selected as the primary endpoint of the study. The data collected after 12 months was compared to the data collected after 5 years. As secondary endpoints, the implant survival rate, MBL and complications, including biological complications such as peri-implant mucositis and peri-implantitis, were evaluated.

### 2.9. Statistical Analysis

The statistical analysis was performed using GraphPad Prism 9 software (GraphPad Software, Inc., Boston, MA, USA). For MBL, the Shapiro–Wilk test analysis was performed, and the distribution was not normal, so the Kruskal–Wallis test was used to check the existence of differences between the means. For the soft-tissue analysis, the Shapiro–Wilk test analysis was performed, and the distribution was normal, so a one-way ANOVA Tukey test was applied; each *p* value was adjusted to account for multiple comparisons, and the family-wise alpha threshold and confidence level was 0.05 (95% confidence interval). For the correlation between the MBL and TKT, the Shapiro–Wilk test was carried out and, since the distribution was not normal, the Spearman’s r correlation was used. All the data were given as the means ± standard deviation (SD). *p* < 0.05 was considered statistically significant.

## 3. Results

The overall survival rate of the 75 Conelog^®^, (Camlog Biotechnologies AG, Basel, Switzerland) implants, 30 connective tissue grafts and 30 Geistlich Mucografts was 100% for all the groups at the 5-year follow-up.

### 3.1. Marginal Bone Loss

**Figure 3:**
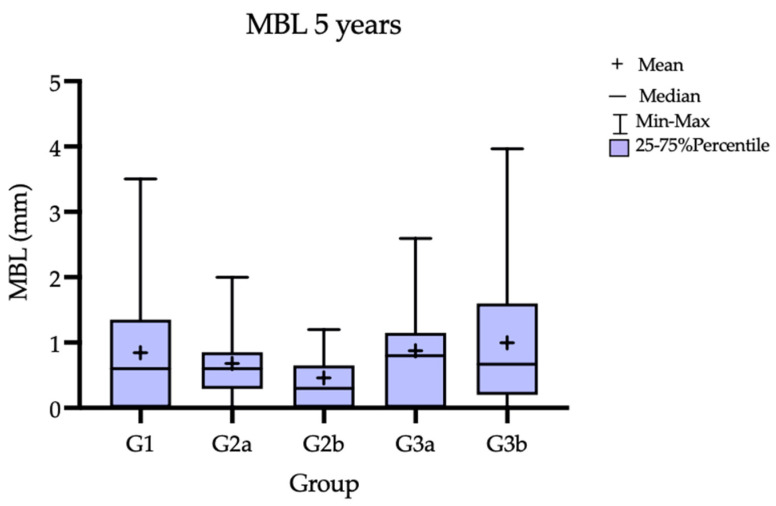
Marginal bone loss after 5 years.

The 12-month results are presented in a previous publication [6]. At the 5-year follow-up, the MBL for all the groups are presented in Table 1 and Figure 3

**Figure 4:**
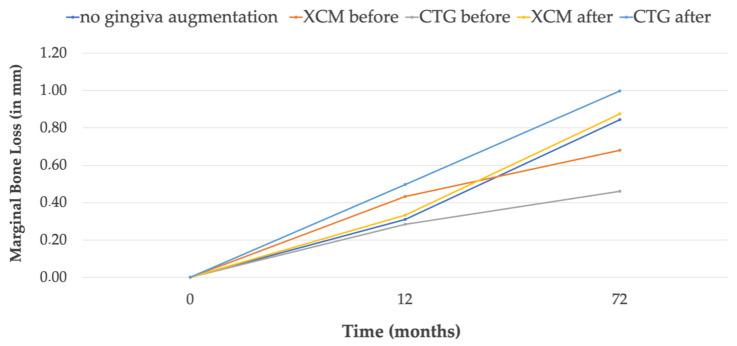
MBL change in individual groups from baseline to 5 years. Simplified chart.

Gradually, progressive bone loss was observed in all the groups throughout the observation period and can be observed on the simplified chart (Figure 4). The lowest MBL, 0.3 mm, was observed in G2b (CTG treatment performed 3 months prior to the implant treatment); the highest MBL, 0.80 mm, was observed in the G3a (CTG treatment performed 3 months after implantation). Nominally better parameters—lower MBL—were obtained for the groups where the soft-tissue surgery was performed 3 months before the implant insertion. However, the differences between the groups were not statistically significant.

### 3.2. Soft-Tissue Volume

**Figure 5:**
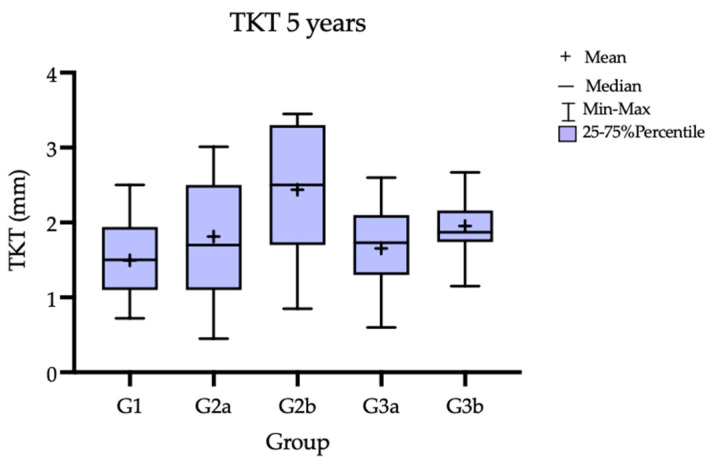
Soft-tissue thickness at 5-year follow-up.

**Figure 6:**
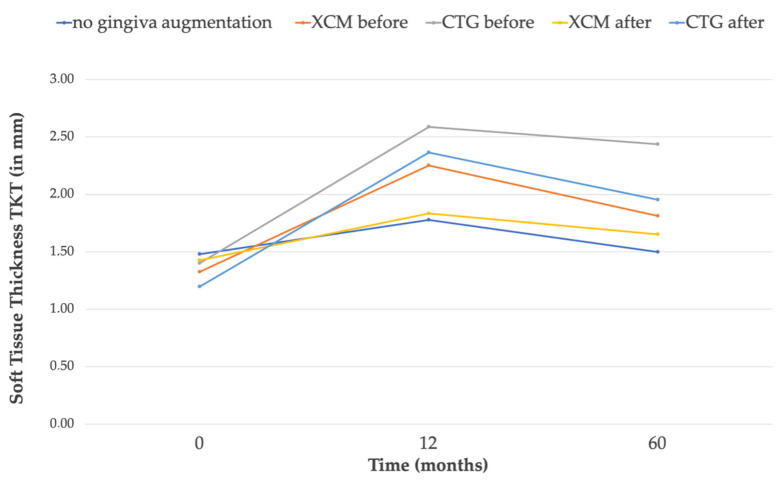
Soft-tissue thickness change for individual groups at the 5-year follow-up. Simplified chart.

**Figure 7:**
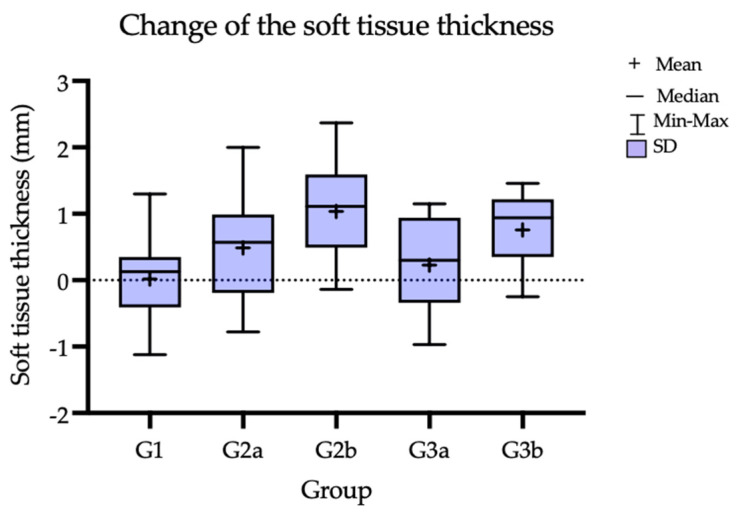
Change of the keratinized soft-tissue thickness (before treatment and at 5 years).

An increase in soft-tissue thickness was observed in all the groups after 12 months, as previously reported [7]. Subsequently, comparing the 12 M and 60 M results, the soft-tissue thickness decreased in all the groups; however, it still maintained a volume above the baseline value (T0). Ultimately, throughout the entire observation period, the greatest increase of 1.035 mm (SD = 0.73 mm) in thickness was observed in G2b (CTG treatment 3 months before implant placement). In both groups where the CTG was used, a greater increase in the soft-tissue thickness compared to the groups with the Geistlich Mucograft was observed after 5 years. In terms of the thickness gain, statistically significant differences were observed for G1 vs. G2b and for G2b vs. G3a and G1 vs. G3b. When comparing the subgroups in terms of the time when the gingival augmentation procedure was performed (3 M pre-implant placement vs. 3 M post-implant placement), better parameters in terms of tissue thickness were observed for the subgroups where the soft-tissue procedure preceded the implantation. The TKT change over time and TKT comparison between the groups are reported in Table 2 and Table 3, and the mean thickness change baseline compared to 60 M in Table 4. Figure 5 presents the TKT after 5 years, Figure 6 shows the trend over the time of observation; Figure 7 presents the mean tissue thickness change—the baseline compared to 60 M.

### 3.3. Correlation between MBL and Keratinized Soft-Tissue Thickness TKT

**Figure 8:**
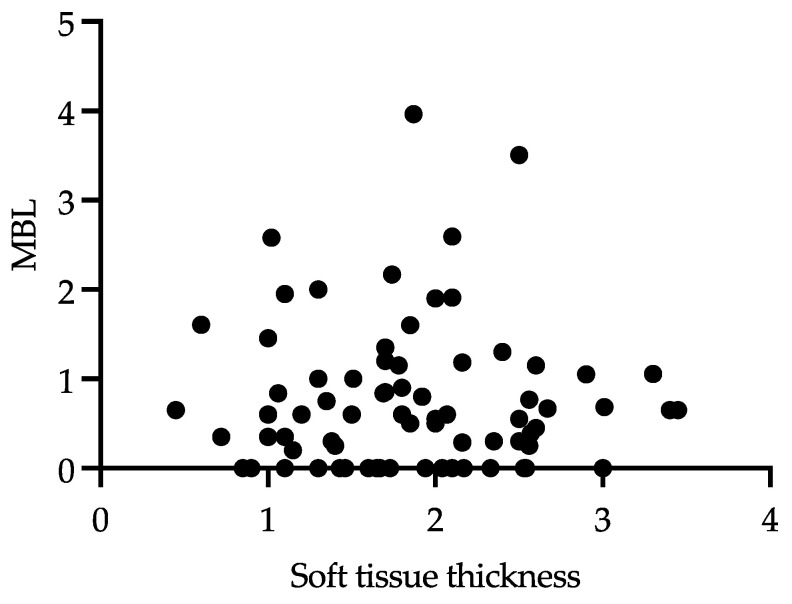
Correlation between MBL and TKT. Shapiro–Wilk test was carried out; since the distribution was not normal, the Spearman’s r correlation was used. r = 0.02662.

It was found that in Group G2b (CTG, before implant placement), a TKT of 2.43 mm was present at the end of the observation period; in the same group, the lowest bone loss, MBL 0.3 mm, was observed. Although this data could suggest that when a lower tissue thickness is present the more MBL around implants could be present, when analyzing all the groups together, considering the relationship of TKT and MBL, there was neither a statistically significant correlation nor inverse correlation found between the MBL and TKT (Figure 8).

### 3.4. Clinical Outcomes

The implant survival rate was 100%. A total number of four implants met the criteria of Derks for peri-implantitis (BOP+ and MBL > 2 mm [8]). This corresponds to 5.3% of the total implants placed in the study. In G2a and G2b, no cases of peri-implantitis were observed during the study period. For the incidence of peri-implantitis, the number of affected implants in each group is presented in Table 5.

## 4. Discussion

Aguido et al. [9] in a 25- to 30-year follow-up study of free gingival grafts in recession treatments found that the gingival phenotype modification achieved by the free gingival graft may prevent the development or progression of non-carious cervical lesions in humans, and concluded that the thickness and width of the attached gingiva plays a major role in this regard. Bertl et al., in the latest systematic review, found a coronally advanced flap and CTG the most predictable in the long-term treatment for root coverage [10]. Earlier reviews presented an insufficient amount of evidence as to the positive effect of the width and height of the peri-implant soft-tissue disease. However, nowadays, there is solid evidence that adequate peri-implant soft-tissue width and keratinized soft-tissue volume have a positive impact on the long-term stability of peri-implant tissues [11,12,13,14,15,16,17].

Various surgical approaches have been presented to increase the soft-tissue volume. Santamaria et al. [18] evaluated the effectiveness of the collagen matrix and xenogeneic acellular dermal matrix associated with the coronally advanced flap technique; they found that both matrices provided similar results in the treatment of single gingival recessions and, additionally, increase the gingival thickness. The Mucograft that was used in our study is a porcine-derived resorbable matrix commonly used as a soft-tissue substitute, avoiding the disadvantages of autologous tissue grafts, such as increased morbidity.

The objective of our study is a long-term perspective on patients treated in our facility comparing CTG to CMX Mucograft. Such long-term observations from an RCT study are lacking in the literature.

Huang et al. found that, despite the superiority of free gingival grafts (FFG) to restore keratinized gingiva, both Mucografts and FGGs can increase soft-tissue volume around dental implants to better maintain the peri-implant health and obtain comparable aesthetic outcomes [19]. Similarly, in our study, we observed that, compared to the control group where no augmentation was done, we have nominally increased the TKT with both the CMX and CTG.

It is known that the volume of biomaterial changes over time. In the latest systematic review by Moraschini [20], it was found that a CMX showed a lower increase in gingival thickness when compared to a CTG. In this case, only short-term observations were qualified. In our study, we found a nominally lower mean increase in volume for the CMX groups (G2a: 0.486 mm and G3a: 0.226 mm) compared to the CTG groups (G2b: 1.035 mm and G3b: 0.76 mm). However, statistically significant differences in soft-tissue thickness gain were found between G1 vs. G2b (no gingival augmentation vs. CTG before), G1 vs. G3b (no augmentation vs. CTG after) and for G2b vs. G3a (CTG before vs. CMX after).

Schmitt et al. compared the peri-implant keratinized mucosa regenerated with the Geistlich Mucograft and FGG. He found that most of the volume loss of the regenerated tissue occurred in the first 90 days post-surgery, and slowed down in both groups but did not completely stop until the end of the observation period (5 years) [21]. Schmitt showed that, after 5 years, the shrinkage of the CMX was significantly greater compared to the FGG [21]. Similarly, in our study, a greater shrinkage was observed for the CMX compared to the CTG in the long-term (12 M compared to 5 Y). However, it should be noted that the thickness was still held above the initial value (T0), and that no statistically significant difference was found between the groups.

Multiple factors might affect the changes in the MBL, such as smoking habits, diabetes and a history of periodontal disease, which is the reason that, in our study, patients with these conditions were excluded. A progressive MBL around the implant neck is the prelude to peri-implantitis development [22]. Gianfilippo et al. [23], in a recent systematic review, found that the soft-tissue thickness is correlated with the MBL, except in cases of platform-switching implants, when implants with thin tissues and screw-retained prostheses are used. In our study, when analyzing the relationship of the TKT and MBL after 5 years of follow-up, there was no correlation found between MBL and TKT. We believe that this may be due to the fact that most of the patients participating in the study had at least 1.49 mm TKT, so there were no patients with a very thin biotype.

Strong evidence exists that cemented restorations may influence MBL due to excessive cement. Therefore, it is advised to use screw-retained restorations that limit the influence of cement on MBL, instead [24,25,26,27]. Strauss et al. [28] also reported on the differences in MBL between cemented and screw-retained restoration. Nevertheless, the differences between the cemented and screw-retained restorations were rated negligible from the clinical point of view. In our study at the time, cemented restorations were the standard treatment protocol.

The meta-analysis of Suárez-López del Amo et al. [29] confirmed that a minimum of ≥2 mm of soft-tissue thickness is required to maintain the bone level, and the higher values of MBL occur in the presence of thin tissue (<2 mm). To minimize early MBL as a consequence of a lack of mucosal thickness, several authors, including Puisys and Linkevicius [30], Wiesner et al. [31], Puzio et al. [6], advise the reestablishment of soft-tissue volume by soft-tissue grafting. Puisys [30] shows that, in the case of both thick and thickened tissues with >2 mm, less MBL can be observed. It is worth noting that, in our study, during the 5 years of follow-up, no implants were lost and only four cases of peri-implantitis (5.3% of all the placed implants) were observed. In our study, we did not record more clinical complications than statistically found in the literature [26,32]. We believe that this may be due to the fact that most of the patients participating in the study had a TKT of at least 1.49 mm at the 5-year follow-up visit.

Many other factors are important for the well-being and stability of the tissues surrounding the implant; attention is paid to the materials of prosthetic components that contact the soft tissue [33].The interesting results of an animal study were recently presented by Kulakov et al. [34]. Kulakov found that the improved treatment effect of collagen matrices can be achieved when bone marrow-derived mesenchymal stromal cells obtained from the subject animal are injected; additionally, they found that the implantation of collagen matrices under the mucoperiosteal flap leads to better augmentation outcomes [34]. Other factors that influence the choice of material are patient morbidity, satisfaction and compliance. The literature [35] and our experience show that soft-tissue augmentation with a Geistlich Mucograft causes significantly less pain during speaking and chewing compared to autogenous tissue harvested from the palate.

Regarding our research hypotheses:

1. The first research hypothesis was rejected. In all the groups, a nominal gain of tissue thickness was observed comparing the baseline to 5 Y post-operation. Statistically significant differences in soft-tissue thickness gain were found between G1 vs. G2b (no gingival augmentation vs. CTG before), G1 vs. G3b (no augmentation vs. CTG after) and for G2b vs. G3a (CTG before vs. CMX after).
2. The second hypothesis of the non-inferiority of the Geistlich Mucograft compared to the connective tissue grafts in the augmentation gingiva could not be verified. Although the CMX material showed good results in comparison to the control, a statistically significant difference to the CTG, in terms of thickness gain, was observed, with the CTG being superior. When comparing the baseline to the 5 Y thickness, the results were ranked in order of the smallest to the largest tissue gain: no augmentation (0.018 mm) < CMX after (0.226 mm) < CMX before (0.486 mm) < CTG after (0.76 mm) < CTG before (1.035 mm).

## 5. Limitations

Despite the groups of 15 implants each, a large standard deviation was observed in the groups. Although nominal differences between the groups were observed, the large standard deviation significantly influenced the statistical analysis of the data and the difficulty in identifying statistically significant differences.

## 6. Conclusions

For both the CTG and Geistlich Mucograft, better clinical parameters were observed, both for maintaining the alveolar bone level and the thickness of the keratinized gingiva, compared to the control group with no gingival augmentation. Performing a gingival augmentation before the implant treatment resulted in an improvement of the clinical outcomes. In our study, we found a nominally lower increase in tissue thickness for the corresponding CMX groups compared to the CTG groups. Statistically important differences in soft-tissue thickness gain during the 5 years of observation were observed for groups G1 vs. G2b (no augmentation vs. CTG before), G1 vs. G3b (no augmentation vs. CTG after) and for groups G2b vs. G3a (CTG before vs. CMX after).

## Figures and Tables

**Table 1:** Marginal bone loss after 5 years. No statistically significant differences were found between the groups (*p* < 0.05).

	Group Description	No Gingiva Augmentation	CMX Before	CTG Before	CMX After	CTG After
	Group	G1	G2a	G2b	G3a	G3b
MBL 5 years (in mm)	Min	0	0	0	0	0
	Max	3.5	2	1.2	2.6	3.7
	Median:	0.60	0.60	0.3	0.80	0.67
	25% Percentile:	0	0.29	0	0	0.2
	75% Percentile:	1.35	0.85	0.65	1.15	1.6
	Range	3.50	2.0	1.2	2.59	3.96

**Table 2:** TKT in mm. Mean T0—initial value, 12 M—12-month value, 60 M—5 year value. Mean change of the soft tissue (Mean T0–Mean 60 M) thickness between T0 and 5 Y in mm.

	Group Description	No Giginva Augmentation	CMX Before	CTG Before	CMX After	CTG After
	Group	G1	G2a	G2b	G3a	G3b
TKT (in mm)	Mean T0	1.48	1.326	1.4	1.426	1.2
	Mean 12 M	1.776	2.25	2.587	1.835	2.36
	Mean 60 M	1.496	1.813	2.436	1.652	1.95
	Mean change T0 vs. 60 M	0.018	0.486	1.035	0.226	0.76

**Table 3:** TKT 60 M. Tukey multiple comparison test between the groups. Differences were observed for groups G1 vs. G2b (no gingival augmentation vs. CTG before) and for groups G2b vs. G3a (CTG before vs. CMX after). ns, non sugificant; ** for a level of significance.

Tukey’s Multiple Comparisons Test	Mean Diff.	95.00% CI of Diff.	Significant	Summary	Adjusted *p* Value
G1 vs. G2a	−0.3167	−0.9843 to 0.3510	No	ns	0.6697
**G1 vs. G2b**	**−0.94**	**−1.608 to −0.2723**	**Yes**	**	**0.0019**
G1 vs. G3a	−0.1560	−0.8237 to 0.5117	No	ns	0.9643
G1 vs. G3b	−0.4567	−1.092 to 0.2110	No	ns	0.3149
G2a vs. G2b	−0.6233	−1.292 to 0.01230	No	ns	0.0782
G2a vs. G2a	0.1607	−0.5070 to 0.8283	No	ns	0.9603
G2a vs. G3b	−0.14	−0.8077 to 0.50277	No	ns	0.9759
**G2b vs. G3a**	**0.784**	**0.1163 to 1.452**	**Yes**	**	**0.0136**
G2b vs. G3b	0.4833	−0.1843 to 1.151	No	ns	0.2606
G3a vs. G3b	−0.3007	−0.9683 to 0.3670	No	ns	0.7108

**Table 4:** Mean change of TKK baseline compared to 60 M. Tukey multiple comparison test between the groups. Differences were observed for groups G1 vs. G2b (no augmentation vs. CTG before), G1 vs. G3b (no augmentation vs. CTG after) and for groups G2b vs. G3a (CTG before vs. CMX after). Ns, non sugificant; *, ** and *** for a level of significance.

Tukey’s Multiple Comparisons Test	Mean Diff.	95.00% CI of Diff.	Significant	Summary	Adjusted *p* Value
G1 vs. G2a	−0.4693	−1.155 to 0.2161	No	ns	0.3180
**G1 vs. G2b**	**−1.018**	**−1.703 to −0.3325**	**Yes**	***	**0.0008**
G1 vs. G3a	−0.2087	−0.8941 to 0.4768	No	ns	0.9130
**G1 vs. G3b**	**−0.7387**	**−1.424 to 0.05319**	**Yes**	*	**0.0284**
G2a vs. G2b	−0.5487	−1.234 to 0.1368	No	ns	0.1769
G2a vs. G2a	0.2607	−0.4248 to 0.9461	No	ns	0.8238
G2a vs. G3b	−0.2693	−0.9548 to 0.4161	No	ns	0.8058
**G2b vs. G3a**	**0.8093**	**0.239 to 1.495**	**Yes**	**	**0.0126**
G2b vs. G3b	0.2793	−0.4061 to 0.9648	No	ns	0.7842
G3a vs. G3b	−0.5300	−1.215 to 0.155	No	ns	0.2052

**Table 5:** Number of implants affected with peri-implantitis in each group.

	No Gingiva Augmentation	CMX Before	CTG Before	CMX After	CTG After
	G1	G2a	G2b	G3a	G3b
Number of implants	2	0	0	1	1
Percentage of implants	13%	0%	0%	6.6%	6.6%

## Data Availability

The data are available upon request from the corresponding author.

## References

[1] Linkevicius T., Apse P., Grybauskas S., Puisys A. (2009). The influence of soft tissue thickness on crestal bone changes around implants: A 1-year prospective controlled clinical trial. Int. J. Oral Maxillofac. Implant..

[2] Bhat P., Thakur S., Kulkarni S. (2015). The influence of soft tissue biotype on the marginal bone changes around dental implants: A 1-year prospective clinico-radiological study. J. Indian Soc. Periodontol..

[3] Lissek M., Boeker M., Happe A. (2020). How thick is the oral mucosa around implants after augmentation with different materials: A systematic review of the effectiveness of substitute matrices in comparison to connective tissue grafts. Int. J. Mol. Sci..

[4] Scharf D.R., Tarnow D.P. (1992). Modified roll technique for localized alveolar ridge augmentation. Int. J. Periodontics Restor. Dent..

[5] Langer B., Calagna L. (1980). The subepithelial connective tissue graft. J. Prosthet. Dent..

[6] Puzio M., Hadzik J., Błaszczyszyn A., Gedrange T., Dominiak M. (2020). Soft tissue augmentation around dental implants with connective tissue graft (CTG) and xenogenic collagen matrix (XCM). 1-year randomized control trail. Ann. Anat..

[7] Puzio M., Błaszczyszyn A., Hadzik J., Dominiak M. (2018). Ultrasound assessment of soft tissue augmentation around implants in the aesthetic zone using a connective tissue graft and xenogeneic collagen matrix—1-year randomised follow-up. Ann. Anat..

[8] Derks J., Schaller D., Håkansson J., Wennström J.L., Tomasi C., Berglundh T. (2016). Peri-implantitis—Onset and pattern of progression. J. Clin. Periodontol..

[9] Agudio G., Chambrone L., Selvaggi F., Pini-Prato G.P. (2019). Effect of gingival augmentation procedure (free gingival graft) on reducing the risk of non-carious cervical lesions: A 25- to 30-year follow-up study. J. Periodontol..

[10] Bertl K., Spineli L.M., Mohandis K., Stavropoulos A. (2021). Root coverage stability: A systematic overview of controlled clinical trials with at least 5 years of follow-up. Clin. Exp. Dent. Res..

[11] Heitz-Mayfield L.J.A. (2008). Peri-implant diseases: Diagnosis and risk indicators. J. Clin. Periodontol..

[12] Hadzik J., Botzenhart U., Krawiec M., Gedrange T., Heinemann F., Vegh A., Dominiak M. (2017). Comparative evaluation of the effectiveness of the implantation in the lateral part of the mandible between short tissue level (TE) and bone level (BL) implant systems. Ann. Anat..

[13] Bassetti R.G., Stähli A., Bassetti M.A., Sculean A. (2016). Soft tissue augmentation procedures at second-stage surgery: A systematic review. Clin. Oral Investig..

[14] Lindhe J., Meyle J. (2008). Peri-implant diseases: Consensus Report of the Sixth European Workshop on Periodontology. J. Clin. Periodontol..

[15] Souza A.B., Tormena M., Matarazzo F., Araújo M.G. (2016). The influence of peri-implant keratinized mucosa on brushing discomfort and peri-implant tissue health. Clin. Oral Implant. Res..

[16] Moraschini V., Luz D., Velloso G., Barboza E.d.S.P. (2017). Quality assessment of systematic reviews of the significance of keratinized mucosa on implant health. Int. J. Oral Maxillofac. Surg..

[17] Hadzik J., Kubasiewicz-Ross P., Nawrot-Hadzik I., Gedrange T., Pitułaj A., Dominiak M. (2021). Short (6 mm) and Regular Dental Implants in the Posterior Maxilla-7-Years Follow-up Study. J. Clin. Med..

[18] Santamaria M.P., Rossato A., Miguel M.M.V., Fonseca M.B., Bautista C.R.G., de Marco A.C., Mathias-Santamaria I.F., Ferreira Ferraz L.F. (2022). Comparison of two types of xenogeneic matrices to treat single gingival recessions: A randomized clinical trial. J. Periodontol..

[19] Huang J.P., Liu J.M., Wu Y.M., Dai A., Hu H.J., He F.M., Chen Q.M., Li X.J., Sun P., Ding P.H. (2021). Clinical evaluation of xenogeneic collagen matrix versus free gingival grafts for keratinized mucosa augmentation around dental implants: A randomized controlled clinical trial. J. Clin. Periodontol..

[20] Moraschini V., Guimarães H.B., Cavalcante I.C., Calasans-Maia M.D. (2020). Clinical efficacy of xenogeneic collagen matrix in augmenting keratinized mucosa round dental implants: A systematic review and meta-analysis. Clin. Oral Investig..

[21] Schmitt C.M., Moest T., Lutz R., Wehrhan F., Neukam F.W., Schlegel K.A. (2016). Long-term outcomes after vestibuloplasty with a porcine collagen matrix (Mucograft^®^) versus the free gingival graft: A comparative prospective clinical trial. Clin. Oral Implant. Res..

[22] Galindo-Moreno P., León-Cano A., Ortega-Oller I., Monje A., O’valle F., Catena A. (2015). Marginal bone loss as success criterion in implant dentistry: Beyond 2 mm. Clin. Oral Implant. Res..

[23] Di Gianfilippo R., Valente N.A., Toti P., Wang H.L., Barone A. (2020). Influence of implant mucosal thickness on early bone loss: A systematic review with meta-analysis. J. Periodontal Implant. Sci..

[24] Wittneben J.G., Joda T., Weber H.P., Brägger U. (2017). Screw retained vs. cement retained implant-supported fixed dental prosthesis. Periodontol. 2000.

[25] Sailer I., Mühlemann S., Zwahlen M., Hämmerle C.H.F., Schneider D. (2012). Cemented and screw-retained implant reconstructions: A systematic review of the survival and complication rates. Clin. Oral Implant. Res..

[26] Fu J.H., Wang H.L. (2020). Breaking the wave of peri-implantitis. Periodontol. 2000.

[27] Krawiec M., Olchowy C., Kubasiewicz-Ross P., Hadzik J., Dominiak M. (2022). Role of implant loading time in the prevention of marginal bone loss after implant-supported restorations: A targeted review. Dent. Med. Probl..

[28] Strauss F.J., Hämmerle C.H.F., Thoma D.S. (2021). Short communication: Cemented implant reconstructions are associated with less marginal bone loss than screw-retained reconstructions at 3 and 5 years of loading. Clin. Oral Implant. Res..

[29] Suárez-López del Amo F., Lin G.-H., Monje A., Galindo-Moreno P., Wang H.-L. (2016). Influence of Soft Tissue Thickness on Peri-Implant Marginal Bone Loss: A Systematic Review and Meta-Analysis. J. Periodontol..

[30] Puisys A., Linkevicius T. (2015). The influence of mucosal tissue thickening on crestal bone stability around bone-level implants. A prospective controlled clinical trial. Clin. Oral Implant. Res..

[31] Wiesner G., Esposito M., Worthington H., Schlee M. (2010). Connective tissue grafts for thickening peri-implant tissues at implant placement. One-year results from an explanatory split-mouth randomised controlled clinical trial. Eur. J. Oral Implantol..

[32] Jepsen S., Berglundh T., Genco R., Aass A.M., Demirel K., Derks J., Figuero E., Giovannoli J.L., Goldstein M., Lambert F. (2015). Primary prevention of peri-implantitis: Managing peri-implant mucositis. J. Clin. Periodontol..

[33] Hadzik J., Kubasiewicz-Ross P., Gębarowski T., Waloszczyk N., Maciej A., Stolarczyk A., Gedrange T., Dominiak M., Szajna E., Simka W. (2023). An Experimental Anodized Titanium Surface for Transgingival Dental Implant Elements&mdash;Preliminary Report. J. Funct. Biomater..

[34] Kulakov A., Kogan E., Brailovskaya T., Vedyaeva A., Zharkov N., Krasilnikova O., Krasheninnikov M., Baranovskii D., Rasulov T., Klabukov I. (2021). Mesenchymal stromal cells enhance vascularization and epithelialization within 7 days after gingival augmentation with collagen matrices in rabbits. Dent. J..

[35] Fu X., Wang Y., Chen B., Tian J., Lin Y., Zhang Y. (2021). Patient-reported outcome measures and clinical outcomes following peri-implant vestibuloplasty with a free gingival graft versus xenogeneic collagen matrix: A comparative prospective clinical study. Int. J. Implant Dent..

